# A Reference‐Free Algorithm Discovers Regulation in the Plant Transcriptome

**DOI:** 10.1002/pld3.70061

**Published:** 2026-04-09

**Authors:** Elisabeth Meyer, Evan V. Saldivar, Marek Kokot, Bo Xue, Sebastian Deorowicz, Seung Y. Rhee, Julia Salzman

**Affiliations:** ^1^ Department of Biochemistry Stanford University Stanford California USA; ^2^ Department of Biomedical Data Science Stanford University Stanford California USA; ^3^ Department of Biology Stanford University Stanford California USA; ^4^ Department of Plant Biology Carnegie Institution for Science Stanford California USA; ^5^ Department of Algorithmics and Software Silesian University of Technology Gliwice Poland; ^6^ Plant Resilience Institute, Department of Biochemistry and Molecular Biology, Department of Plant Biology, Department of Plant, Soil, and Microbial Sciences Michigan State University East Lansing Michigan USA; ^7^ Department of Statistics (by courtesy) Stanford University Stanford California USA; ^8^ Department of Biology (by courtesy) Stanford University Stanford California USA

**Keywords:** genomics, reference‐free, transcriptomics

## Abstract

Most plant genomes and their (post‐)transcriptional regulation remain unknown. We used SPLASH—a new, reference genome‐free sequence variation detection algorithm—to analyze transcriptional and post‐transcriptional regulation from RNA‐seq data. We discovered allelic variation in expression during maize pollen development and imbibition‐dependent cryptic splicing in Arabidopsis seeds. SPLASH enables discovery of novel regulatory mechanisms, including differential regulation of genes from parental haplotypes of hybrids, without the use of alignment to a reference genome.

The study of plant genomes and transcriptomes is fundamental to advancing basic biological science, crop resilience, and ecosystem stewardship. Today, plant genomic analysis typically begins with alignment to a reference genome or transcriptome. However, alignment‐based approaches are limited by how well aligners perform and how well the available reference genome approximates the true genome. The assembly of plant genomes is particularly challenging due to complexities such as intrinsic plasticity, high fractions of repetitive sequence (Heslop‐Harrison and Schwarzacher 2011; Gaut et al. 2000), polyploidy, and gene duplications (Sun et al. 2022; Clark and Donoghue 2018).

These problems, while acute in plants, are a general obstacle to discovery across the tree of life. To address them, we recently introduced a new approach to analyze regulation of genomes and transcriptomes using an ultra‐efficient, reference‐free, statistical approach called SPLASH (Chaung et al. 2023; Kokot et al. 2024). In brief, SPLASH identifies statistically significant sample‐specific sequence variation directly from raw reads, bypassing alignment to a reference (Chaung et al. 2023; Kokot et al. 2024). From raw FASTQ reads, SPLASH records pairs of contiguous sequences of a set length (i.e. pairs of kmers) where the first sequence is constant and the second sequence is variable. The constant kmer is called an “anchor”, and the variable kmers that follow are “targets” associated with that anchor. SPLASH then identifies anchors whose associated targets vary in relative abundance among samples. If an anchor’s targets' relative abundance is statistically significantly different between experimental samples, then the anchor is deemed significant. SPLASH can detect numerous biological processes that diversify transcript sequence, including alternative splicing and differential expression of homologs (Figure S1A; Supporting Information: Methods). SPLASH can also identify sequence variation in organisms co‐associating with the host. The significant anchors and their associated variant targets can be used to investigate the mechanism behind the variation (Figure S1B). A reference genome or sequence database can be used downstream to add annotation such as a gene identity, but the initial discovery is not limited by the availability or quality of a reference genome.

To illustrate the power of SPLASH to discover (post‐)transcriptional regulation in plants, we re‐analyzed four RNA‐seq datasets from maize ( *Zea mays* ), *Arabidopsis thaliana* , and sorghum ( *Sorghum bicolor* ) (Figure 1; Figure S1). SPLASH makes thousands of discoveries in each dataset (Supporting Information) without information about sample identity or reference genomes (Supporting Information: Methods).

**FIGURE 1:**
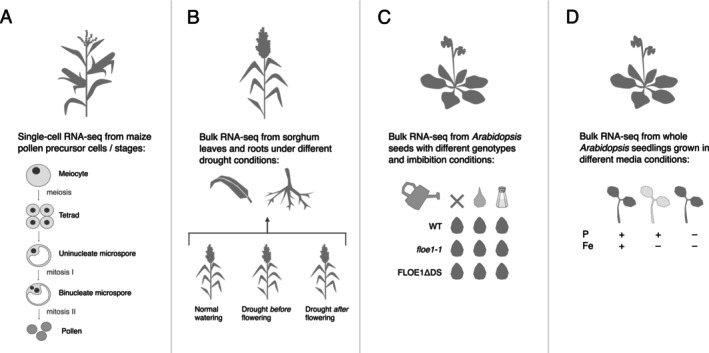
Overview of datasets analyzed with SPLASH in this paper. (A) A single‐cell study of maize pollen precursors. (B) A bulk RNA‐seq study of sorghum under different drought conditions. (C) A bulk RNA‐seq study of *Arabidopsis* seed germination, with wild‐type and mutant strains in the FLOE1 gene, in conditions of no water, plain water imbibition, or salt water imbibition. (D) A bulk RNA‐seq study of *Arabidopsis* seedlings under phosphorus (P), iron (Fe), or iron/phosphorus (Fe/P) deficiency. *floe1‐1* = knock‐out mutation of FLOE1; FLOE1ΔDS = aspartic acid‐serine‐rich disordered domain deletion mutation of FLOE1; WT = wild type FLOE1.

## 1 SPLASH Discovers Complex Transcript Abundance Patterns Associated With Plant Genes

**FIGURE 2:**
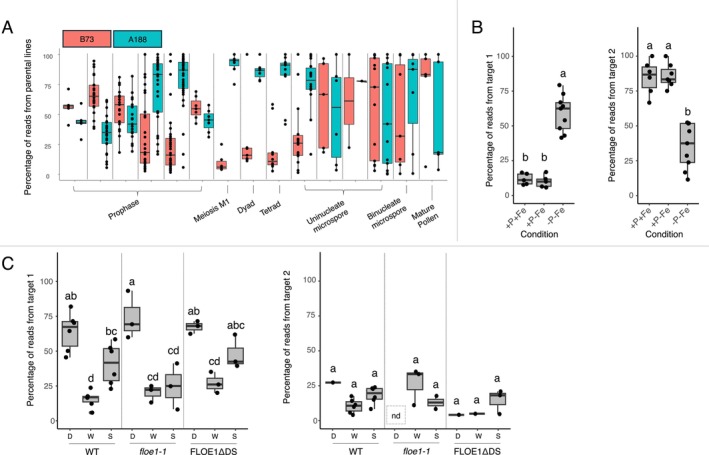
Percentage of reads within each sample from each of the top two targets, calculated as number of reads from that particular target divided by total number of reads from the anchor. The top two targets typically represent the majority of reads even though most anchors have more than two targets. Horizontal lines in the boxes represent median; individual datapoints represent individual samples (single cells for maize pollen). For (B) and (C), conditions were compared for each target using an ANOVA and Tukey's test; the results are summarized by compact letter display (Supporting Information: Methods). “ND” indicates no reads were found for a particular target within a particular sample. (A) Maize pollen dataset: the top two targets for this anchor align to alleles of Zm00001eb173470 from B73 and A188. The differential expression of these alleles varies by pollen stage. The steps within the prophase label are, in order: leptotene with peripheral nucleolus, leptotene with central nucleolus, prezygotene, zygotene, pachytene, and interphase. *Note:* This anchor was only found in 260 out of 642 samples. (B) *Arabidopsis* iron/phosphorus deprivation dataset: target 1 maps to a splice junction in AT1G74270 (ribosomal protein EL33Y), while target 2 includes the intron. ‐P‐Fe indicates the phosphorus and iron doubly deprived condition; +P‐Fe is only iron deprivation; and +P+Fe indicates no deprivation. (C) *Arabidopsis* FLOE1 dataset: target 1 maps to an annotated splice junction between exons in AT2G36720, but target 2 maps to a cryptic splicing event from inside an intron to an exon. WT (wild type), *floe1‐1* (FLOE1 deletion mutant), and FLOE1ΔDS (FLOE1 mutant with N‐terminal disordered region deleted) indicate the different seed genotypes; D (dry), W (wet), and S (salt) indicate the imbibition conditions for the seeds.

We re‐analyzed a single‐cell RNA‐seq study of maize pollen precursors from a hybrid line derived from a cross between inbred lines B73 and A188 (Nelms and Walbot 2022). In this dataset (Nelms and Walbot 2022), we found 8,989,404 significant anchors. SPLASH discovered developmentally regulated genes across both parental alleles, without requiring alignment to a reference genome. For example, the A188 allele of Zm00001eb173470 (preliminary annotation ID: Zm00001e021816), which encodes a ribosome‐like protein, is expressed more highly than the B73 allele during meiosis M1 (Figure 2A, Figure S2A). However, the B73 allele is more dominantly expressed than the A188 allele in other stages of pollen development such as prophase leptotene stages. In this example, the SPLASH algorithm detected that two closely related sequences were expressed differently throughout pollen development. Matching these two sequences back to the parental alleles can either be achieved by querying a sequence database, such as GenBank, or by aligning to reference genomes. Thus, the reference genome is useful for biological interpretation but not necessary for the initial discovery of regulated expression. The complex developmental regulation of this gene, found by SPLASH without using either of the reference genomes, underscores the simplicity of a reference‐free approach for gene‐regulation discovery in plants.

In addition, we re‐analyzed a study of wild type *Arabidopsis* seedlings, which showed that chlorosis (loss of chlorophyll) induced by iron deficiency involves a phosphorus‐dependent pathway in which doubly‐deprived plants in iron and phosphorus stay green (Nam et al. 2021). However, the genetic pathway that underpins this phenotype is not fully elucidated, warranting further study. A snapshot of SPLASH's findings includes condition‐dependent alternative splicing in gene AT1G74270 (ribosomal protein EL33Y): the phosphorus‐deficient samples predominantly express the spliced isoform (target 1), whereas the samples not deprived of phosphorus predominantly express an intron‐retaining version (target 2; Figure 2B, Figure S2B). In this example, the gene was identified by aligning the SPLASH‐identified sequences to the *Arabidopsis* genome where the splice junctions were already annotated (Supporting Information: Methods). To our knowledge, this is the first example of splicing regulation in a plant ribosomal protein gene impacted by nutrient deprivation.

Finally, we re‐analyzed a study of *Arabidopsis* that discovered the gene FLOE1 as a regulator of seed germination (Dorone et al. 2021). This study showed that FLOE1 controls seed germination under water‐limiting conditions and senses water availability through condensate formation. Condensate formation is important for FLOE1's ability to control germination. How FLOE1's condensate formation controls germination remains unknown. The study includes RNA‐seq from seeds of wild type, FLOE1 deletion (*floe1‐1*), and the aspartic acid and serine (DS)‐rich intrinsically disordered region deletion (FLOE1ΔDS) plants, which were dry, imbibed with plain water, or imbibed with salty water (Dorone et al. 2021). The ~6000 significant anchors found in this dataset include imbibition‐induced cryptic splicing in AT2G36720, an acyl‐CoA N‐acyltransferase with RING/FYVE/PHD‐type zinc finger domain‐containing protein, with a splice junction from inside an intron to the 5′ boundary of the adjoining exon identified by aligning the sequences to the reference genome (Figure 2C, Figure S2C). Dry and salt water‐imbibed wild type and FLOE1ΔDS seeds express more of the canonical isoform (target 1) compared to plain water‐imbibed wild type seeds. However, target 1 level is significantly reduced in *floe1‐1* mutants compared to the other genotypes when imbibed with salt water. All conditions, except the dry condition in *floe1‐1*, express a low level of the cryptic splice isoform (target 2). This condition‐dependent splicing implies the possibility of yet‐to‐be discovered imbibition‐dependent splicing in the seed and its effect on protein function.

In summary, SPLASH provides a highly efficient reference‐free approach to detect multiple forms of sample‐specific transcript diversification in plants. Here, we studied several species with assembled genomes to evaluate this analytic framework. However, SPLASH is not limited to the study of well‐characterized species. SPLASH discovers regulated expression and splicing differences directly from sequencing data, without the need for alignment. Although a reference genome can be helpful to identify the genes involved, SPLASH results can be interpreted on the basis of sequence similarity alone (either at the RNA or protein level). We foresee that more unbiased and high‐throughput analysis of plant genomes will allow the plant genomics community to rapidly analyze genetic data from any plant, including those never before studied, without the tedious and time‐consuming steps of genome assembly and alignment.

## 2 Supplementary Information

### 2.1 Total Calls by SPLASH

To find additional examples of biologically interesting sequence variation with SPLASH, we examined unaligned variant targets whose expression varied by sample metadata such as experimental condition or developmental stage. First, we identified “unaligned” anchors, that is, anchors for which one of the top two targets could not be aligned to the genome by STAR. Separately, we applied a generalized linear model (GLM) to all anchors to identify metadata‐regulated anchors (Supporting Information: Methods). Then, we looked for the anchors that were both “unaligned” to the genome as well as metadata‐regulated and investigated these resulting anchors, starting with those having the largest effect size.

In a dataset of field‐droughted sorghum (Varoquaux et al. 2019), there were 11,501 significant anchors (out of 544,476 total significant anchors or 2.11%) for which one of the top two targets did not align to the genome. From the GLM output, there were 10,567 anchors with sample‐dependent variation, corresponding to 3829 unique genes (Table S1). GO enrichment analysis on these regulated genes revealed 77 significantly enriched biological processes, with the top enriched terms being “RNA splicing, via transesterification reactions with bulged adenosine as nucleophile” (GO:0000377) and “hydrogen peroxide catabolic process” (GO:0042744). Of the regulated anchors in sorghum, 408 (3.86%) were also in the unaligned group (Table S2).

**FIGURE 3:**
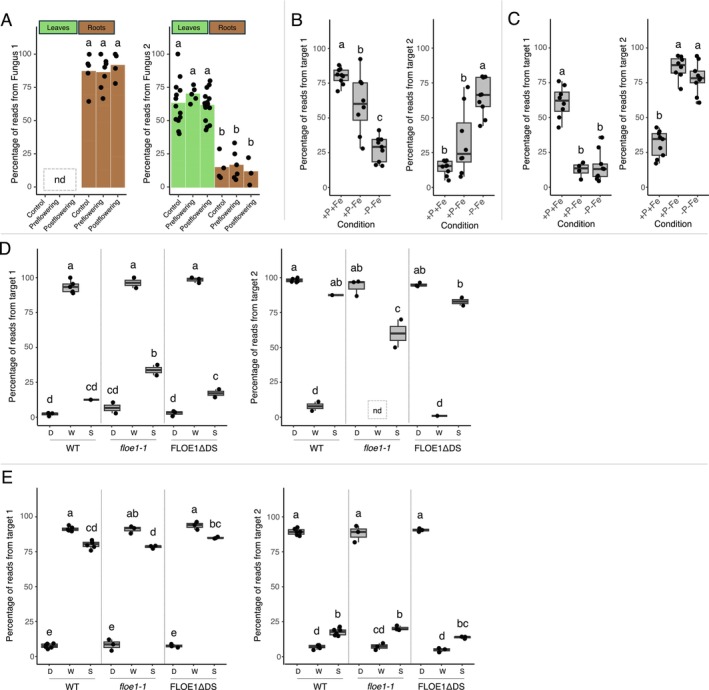
Proportion of reads within each sample from each of the top two targets, calculated as number of reads from that particular target divided by total number of reads from the anchor. The top two target sequences typically represent the majority of reads even though most anchors have more than two targets. Bar height represents the mean and horizontal line in boxes represent the median; individual datapoints represent individual samples. Whether variation depends on experimental condition was tested in two different ways. For (A), we used a generalized linear model to test whether the experimental condition/developmental stage could be predicted from the proportion of targets (Supporting Information: Methods). For (B)–(E), conditions were compared for each target using an ANOVA and Tukey's test; the results are summarized by compact letter display (Supporting Information: Methods). “ND” indicates no reads were found for a particular target within a particular group. (A) Sorghum drought dataset: the top two targets for this anchor BLAST to different fungal species. The most abundant target had the best BLAST hits to fungal species in the genus *Alternaria*; the second most abundant target had the best BLAST hits to species in the genera *Pseudogymnoascus* and *Fusarium* . The abundance of each target differs by tissue type (indicated by bar color). “Control” indicates samples with no drought stress; “preflowering” samples were droughted before the flowering stage; and “postflowering” samples were droughted after the flowering stage. This anchor was only found in 86 out of 198 total samples. (B) *Arabidopsis* iron/phosphorus dataset: target 1 and target 2 for this anchor align to homologous genes AT3G08720 (protein kinase 19) and AT3G08730 (protein‐serine kinase 6) respectively; their relative expression varies by metadata condition. ‐P‐Fe indicates the phosphorus and iron doubly deprived condition; +P‐Fe is only iron deprivation; and +P+Fe indicates no deprivation. (C) *Arabidopsis* iron/phosphorus dataset: target 1 and target 2 for this anchor align to homologous genes AT1G62810 (copper amine oxidase 2) and AT3G43670 (copper amine oxidase 1), respectively; their relative expression varies by metadata condition. (D) *Arabidopsis* FLOE1 dataset: the first and second most abundant targets for this anchor align to homologous squalene monooxygenase genes, AT5G24160 and AT5G24150; their relative expression varies by imbibition condition. WT (wild type), *floe1‐1* (FLOE1 deletion mutant), and FLOE1ΔDS (FLOE1 mutant with N‐terminal disordered region deleted) indicate the different seed genotypes; D (dry), W (wet), and S (salt) indicate the imbibition conditions for the seeds. Note: this anchor was only found in 28 out of 36 samples. (E) *Arabidopsis* FLOE1 dataset: the first and second most abundant targets for this anchor align to homologous ERF/AP2 transcription factors, AT1G78080 (RAP2.4) and AT1G22190 (RAP2.4D); their relative expression varies by imbibition condition.

One of the highest effect‐size metadata‐regulated anchors with one top target unaligned in the sorghum drought dataset had BLAST hits to fungal transcripts (see Table S2 for full list of such anchors). All of the fungal transcripts were identified by sequence similarity using BLAST; in this case, there is no single reference genome that would allow the identification of transcripts from several different species. The most abundant target had the best BLAST hits (with at least 96% coverage and 98.08% identity) to uncharacterized genes from *Alternaria* species (“Fungus 1” in Figure 3A, Figure S3A), which are common plant pathogens (Schmey et al. 2024). For the second most abundant target from this anchor, which was more abundant in leaves compared to roots, the best BLAST hits (all with 100% coverage and 94.44% identity) were annotated as hypothetical proteins from *Pseudogymnoascus verrucosus* and *Fusarium culmorum*, a wheat and sorghum pathogen (Wagacha and Muthomi 2007), or *Fusarium graminearum*, a common fungal pathogen to plants (Lipps et al. 2025) (“Fungus 2” in Figure 3A, Figure S3A).

In the maize pollen dataset (Nelms and Walbot 2022) described in the main text, there were 551,639 anchors (out of 8,989,404 total significant anchors or 6.14%) for which one of the top two targets did not align to the maize genome (B73v5). Some of these unaligned variant targets can be explained by the mismatch between the maize hybrids used to generate the data (A188xB73) and the maize genome used for alignment (B73v5). From the GLM output, there were 190 anchors, corresponding to 78 unique genes, with pollen‐stage‐specific expression (Table S1). No Gene Ontology biological processes were significantly enriched. Five (2.63%) of the expression‐ or condition‐dependent anchors were also in the unaligned group (Table S2).

In the *Arabidopsis* iron/phosphorus deprivation dataset (Nam et al. 2021) described in Section 1, there were 134 anchors (out of 33,650 significant anchors or 0.4%) for which one of the top two targets did not align to the reference genome. From the GLM output, there were 77 anchors with experimental condition‐dependent variation, corresponding to 52 unique genes (Table S1). There were no significantly enriched biological processes. There was no overlap between the regulated anchors (as detected by the GLM approach) and the unaligned anchors.

In the *Arabidopsis* FLOE1 dataset (Dorone et al. 2021) described in Section 1, there were 651 significant anchors (out of 224,642 total significant anchors or 0.29%), for which one of the top two targets did not align to the reference genome. From the GLM output, there were 6470 sequences with condition‐ or genotype‐specific expression, corresponding to 1324 unique genes (Table S1). GO enrichment analysis on the 1324 significant condition‐regulated genes produced 133 significantly enriched biological processes. The most enriched processes were “glutamate biosynthetic process” (GO:0006537), “regulation of developmental vegetative growth” (GO:1905613), “positive regulation of chlorophyll biosynthetic process” (GO:1902326) and “mRNA splice site selection” (GO:0006376). From the expression‐ or condition‐dependent anchors, 11 (0.2%) were also in the unaligned group (Table S2).

In all the datasets, there were also many significant anchors for which both of the top two targets could not be aligned to the genome (712 sequences in the *Arabidopsis* iron/phosphorus dataset; 46,932 in the *Arabidopsis* FLOE1 data; 131,618 in the sorghum data; and 1,099,637 in the maize data). For these anchors, the lists of sequences were so long that we could not query all the sequences by BLAST in an efficient way; for this reason, we chose to investigate the category of anchors for which only one of the top two targets did not align to the reference genome (as described above). As we only investigated a small subset of anchors in depth, we expect there are many more examples of biologically important unaligned targets beyond the ones described in our study.

### 2.2 Additional Discoveries From Pairwise Tests of Metadata Dependence

The GLM approach described above to detect condition‐dependent targets is conservative because condition‐dependent variation may not be apparent from considering only the top two targets. To supplement the GLM approach, we used a second approach: we selected the top 500 anchors with the largest effect sizes in each dataset, and then tested whether the proportion of the top target for these anchors was significantly different between metadata groupings such as experimental condition, genotype, tissue, or developmental stage (Supporting Information: Methods).

In the *Arabidopsis* iron/phosphorus deprivation dataset (Nam et al. 2021), we discovered multiple cases of homologs that are differentially regulated. For homologous genes AT3G08720 (*RIBOSOMAL S6 KINASE 2*, *S6K2*) and AT3G08730 (*RIBOSOMAL S6 KINASE 1*, *S6K1*), relative expression of the homologs depended on phosphorus and iron availability. In samples grown in the presence of phosphorus and iron, ~70% of reads with this anchor correspond to expression of AT3G08720 (target 1), while in samples deficient of both phosphorus and iron, ~70% of the reads with this anchor originate from AT3G08730 (target 2; Figure 3B, Figure S3B). Another example is Copper Amine Oxidase: for samples grown in the presence of iron, ~75% of reads with this anchor correspond to expression of AT1G62810 (copper amine oxidase 2; target 1), while for iron‐deficient samples, ~75–90% of reads with this anchor originate from AT3G43670 (copper amine oxidase 1; target 2) (Figure 3C, Figure S3C). Differential regulation of homologs might indicate rapid evolutionary adaptations occurring in response to nutrient deprivation.

We also discovered condition‐dependent homolog expression in the *Arabidopsis* FLOE1 study (Dorone et al. 2021). For example, squalene monooxygenase genes, AT5G24160 and AT5G24150, are expressed differentially depending on imbibition conditions: imbibition with plain water induces the expression of AT5G24160 (target 1), while dry and salt water conditions result in expression of AT5G24150 (target 2; Figure 3D, Figure S3D). For two homologs in the ERF/AP2 transcription factor family, AT1G78080 (RAP2.4) and AT1G22190 (RAP2.4D), the relative expression varies by whether the samples received any water. Imbibed samples express primarily AT1G78080 (target 1), while dry samples predominantly express AT1G22190 (target 2) (Figure 3E, Figure S3E). As with the case of iron/phosphorus deprivation, the differential homolog expression in response to drought suggests a recent evolutionary adaptation to stress conditions.

Additionally, in the *Arabidopsis* FLOE1 dataset, we discovered regulated alternative splicing in the gene AT5G65080 (MAF5), which has been implicated in flowering timing in response to cold (Ratcliffe et al. 2003). Samples from the dry condition tend to have the final intron spliced out, whereas salt and normal imbibition samples tend to include the final intron sequence (data not shown).

### 2.3 Additional Discoveries of Cryptic Splicing

Because SPLASH does not depend on using metadata, it can be used to discover variation that would not be found when looking for differences between experimental groups. For example, some samples in a dataset may express unannotated splicing isoforms, and SPLASH can discover these novel isoforms even if their expression did not differ by experimental condition. To find examples of splicing that were called significant by SPLASH, we aligned the significant anchors to the genome, and selected anchors with at least one splice junction among the associated variant targets. From this list, we prioritized cases where the variable sequences are the most dissimilar (Supporting Information: Methods).

In the sorghum drought dataset, we detected unannotated splice junctions in genes SORBI_3002G381700 and SORBI_3003G250500 that encode hypothetical proteins, revealing splicing regulation in these poorly studied genes (Meyer et al. 2025).

In the maize pollen dataset, we revealed unannotated splicing in the hypothetical protein Zm00001eb346620 (Meyer et al. 2025). In the *Arabidopsis* iron/phosphorus deprivation dataset, we found anchors where the different targets correspond to unspliced or spliced versions of the transcripts, for example in genes AT3G50480 (a homolog of RPW8, which is a disease resistance gene), AT2G47060 (cytosolic ABA receptor kinase 3), and pseudogene AT1G79245 (Meyer et al. 2025).

For an anchor in the *Arabidopsis* FLOE1 dataset, the associated target sequences have a spliced alignment (aligning in two separate parts) upstream of the gene AT5G01530 into the 5′ untranslated region (UTR) of the gene (Meyer et al. 2025). AT5G01530, also known as LHCB4.1, is part of the light‐harvesting complex (de Bianchi et al. 2011). Splicing upstream of the annotated 5’UTR region could indicate an incomplete gene annotation, with splicing occurring within the 5’UTR (Chung et al. 2006). However, these reads could also be explained by the presence of an upstream open reading frame (uORF), which could have strong regulatory effects such as translation inhibition (von Arnim et al. 2014). In either case, regulated splicing upstream of the LHCB4.1 locus is likely to affect the resulting abundance of the LHCB4.1 protein. We also found cases of splice junctions in introns, for example, in genes AT1G60900 (a putative U2A65 splicing factor involved in flowering regulation) and AT1G80570 (an RNI‐like superfamily protein) (Meyer et al. 2025).

### 2.4 Validation of Previously Unannotated Splice Junctions

In the *Arabidopsis* iron/phosphorus deprivation dataset, we also found instances of unannotated alternative splicing with a low effect size, indicating that there is not a large difference in which the isoforms are expressed in different samples (effect size < 0.053). AT1G13609, a defensin‐like (DEFL) protein known to be regulated by iron deficiency has unannotated splice junctions from within the final exon to downstream of the annotated 3′ UTR end (Figure S4A). We validated this unexpected splicing with amplicon sequencing of the region from PCR‐amplified cDNA (Supporting Information: Methods). The predicted alternative splicing of AT1G79245 was similarly validated (Figure S4B–E; Supporting Information: Methods).

In summary, by bypassing reference alignment, SPLASH reveals complex regulation of sequence diversification mechanisms, including alternative splicing and differential homolog expression. SPLASH represents a critical step forward in plant genomics—here applied to RNA, but also applicable to DNA—that enables rapid, precise discovery of genomic regulation and functional prioritization without an alignment approach.

## 3 Methods

### 3.1 SPLASH Runs

The implementation of SPLASH used in this paper is described in (Kokot et al. 2024). Specifically, SPLASH version 1.9.0 (https://github.com/refresh‐bio/SPLASH/tree/archive/1.9.0) was run on each dataset. When paired end reads were available, only R1 reads (i.e., the first read from each pair of reads) were used as input for SPLASH. This was done because SPLASH does not currently have a way to process paired end reads. For the sorghum drought dataset, only samples from the BTX642 genotype were used as input for SPLASH. For the other datasets, all samples were used as input for SPLASH.

The same following parameters were used for each SPLASH run: n_bins = 128; max_pval_rand_init_alt_max_for_Cjs = 0.1; anchor_len = 27; target_len = 27; gap_len = 0; poly_ACGT_len = 8; anchor_unique_targets_threshold = 1; anchor_count_threshold = 50; anchor_samples_threshold = 1; anchor_sample_counts_threshold = 5; n_most_freq_targets = 10; generate_alt_max_cf_no_tires = 10; altMaximize_iters = 50; train_fraction = 0.25; kmc_use_RAM_only_mode = True; calculate_stats = True; without_SVD = True; with_effect_size_cts = False; enable_pvals_correction = True; fdr_threshold = 0.05.

An example of running the command looks like the following “docker run ‐v `pwd`:/home/ubuntu ghcr.io/refresh‐bio/splash:1.9.0 splash ‐‐n_threads_stage_1 3 ‐‐n_threads_stage_2 8 ‐‐n_bins 128 ‐‐gap_len 0 ‐‐calculate_stats ‐‐dump_Cjs ‐‐n_most_freq_targets 10 ‐‐pvals_correction_col_name pval_rand_init_alt_max ‐‐enable_pvals_correction ‐‐without_SVD ‐‐clean_up ‐‐kmc_use_RAM_only_mode input.txt”. The parameters “‐‐n_threads_stage_1” and “‐‐n_threads_stage_2” determine the number of threads used in each stage based on the memory usage of the species. “Input.txt” is a space‐delimited file that maps the input samples' names to their file paths.

The SPLASH output consists of significant sequences (“anchors”) and the associated diversified sequences (“targets”). SPLASH also automatically outputs some summary statistics including effect size, number of unique targets per anchor, average Hamming distance between each target and the top target.

### 3.2 Local Assembly of Anchors

Local assemblies based on each anchor were generated from reads using the method described in (Henderson et al. 2024).

### 3.3 Filtering out Molecular Biology Artifacts

To remove false positive anchors that originate from sequences present in molecular biology tools, such as sequencing adapters, we used Bowtie2 to align anchors against indices generated from the UniVec database (obtained from ftp://ftp.ncbi.nlm.nih.gov/pub/UniVec/) and a set of Illumina adapters. If an anchor aligned to either database, it was discarded as an artifact.

### 3.4 Alignment of Anchor/Target Sequence to Genome

All reference genome FASTA and BED files were downloaded from Ensembl Plants. For Arabidopsis, we aligned the anchor and target sequences to the TAIR10 assembly (https://ftp.ensemblgenomes.ebi.ac.uk/pub/plants/release‐56/fasta/arabidopsis_thaliana/); for sorghum, we aligned to the NCBIv3 assembly (https://ftp.ensemblgenomes.ebi.ac.uk/pub/plants/release‐56/fasta/sorghum_bicolor/); and for maize, we aligned to the B73 AGPv5 assembly (zeaMay_b73_v5; https://ftp.ensemblgenomes.ebi.ac.uk/pub/plants/release‐56/fasta/zea_mays/).

The alignment and gene name assignment approach was adapted from (Dehghannasiri et al. 2022). For each significant anchor in each dataset, we concatenated the anchor and each target sequence for up to 10 targets per anchor reported by SPLASH, and saved all these concatenated sequences as FASTA files. Then, we aligned each concatenated anchor/target sequence to the respective plant genome using STAR version 2.7.5a. This is STAR command that was used for alignment:

- STAR ‐‐runThreadN 4 ‐‐genomeDir <star_index> ‐‐readFilesIn <concatenated_anchor_target_fasta_file> ‐‐outFileNamePrefix <output_folder> ‐‐twopassMode Basic ‐‐alignIntronMax 1000000 ‐‐chimJunctionOverhangMin 10 ‐‐chimSegmentReadGapMax 0 ‐‐chimOutJunctionFormat 1 ‐‐chimSegmentMin 12 ‐‐chimScoreJunctionNonGTAG ‐4 ‐‐chimNonchimScoreDropMin 10 ‐‐outSAMtype SAM ‐‐chimOutType SeparateSAMold ‐‐outSAMunmapped None ‐‐clip3pAdapterSeq AAAAAAAAA ‐‐outSAMattributes NH HI AS nM NM

Gene names were assigned by extracting exon positions from the STAR BAM output and applying the bedtools function “intersect” to the exon positions as well as a reference BED file of gene and exon boundaries.

To assign anchors to genes/transcripts, the anchors were aligned using Bowtie2 to the respective reference genome.

### 3.5 Querying BLAST

Anchor and target sequences were concatenated and saved as FASTA files. We submitted each FASTA file as a query to BLAST using the following command:

blastn ‐outfmt 6 qseqid sseqid pident length mismatch gapopen qstart qend sstart send evalue bitscore sseqid sgi sacc slen staxids stitle ‐query <fasta_path> ‐remote ‐db nt ‐out <blast_output> ‐evalue 0.2 ‐task blastn ‐dust no ‐word_size 24 ‐reward 1 ‐penalty ‐3 ‐max_target_seqs 20

The BLAST hits were sorted by increasing E‐value, so the “top” hit is the one with the smallest E‐value.

For A188 and B73 allele confirmation, MaizeGDB BLAST (https://maizegdb.org/popcorn/search/sequence_search/home.php?a=BLAST_UI) was used to perform anchors+target BLAST against A188 and B73 genome sequences.

### 3.6 Generalized Linear Model (GLM) to Detect Metadata‐Dependent Target Usage

To automatically detect anchors whose target usage (variant expression) varies depending on the metadata grouping, we used the R package “glmnet” to run a GLMnet Lasso multinomial regression. If the counts from the top two targets of an anchor can predict metadata category, that is, the largest GLM coefficient is greater than one, then the anchor is classified as being metadata‐dependent.

We began by identifying “unaligned” anchors, that is, anchors for which one of the top two targets had a concatenated anchor/target sequence that could not be aligned to the genome by STAR. We also applied the GLM to all anchors to identify metadata‐regulated anchors. Finally, we intersected the group of “unaligned” anchors with the group of metadata‐regulated anchors, and investigated the resulting anchors starting with those having the largest effect size.

### 3.7 Filtering High‐Effect Anchors in *Arabidopsis* FLOE1 and Maize Pollen

To find examples of anchors with condition‐dependent target usage, we initially ranked anchors by decreasing effect size. However, for the *Arabidopsis* FLOE1 and maize pollen datasets, most of the anchors with the highest effect sizes were only present in low numbers of reads. To keep only anchors with robust representation in the raw reads, we required that at least seven of the concatenated anchor/target sequences from the top 10 targets aligned to the genome. We then filtered for only anchors present in at least 500 reads. Finally, we ranked anchors by decreasing effect size and selected the top 500 anchors to inspect for condition‐dependent target usage.

### 3.8 Testing Whether the Fraction of the Top Target Is Significantly Different by Metadata Category

To find examples of anchors with condition‐dependent target usage from the lists of high effect‐size anchors, we began by visually inspecting plots showing expression of each target as grouped by metadata category. For anchors that appeared to have metadata‐dependent target usage, we ran an ANOVA and Tukey's test (using functions aov and TukeyHSD respectively, from the R package “stats”) comparing the fraction of reads from the top targets among different metadata conditions. All the ANOVAs performed were one‐way ANOVAs. For each dataset tested, the metadata groupings were combined into one variable. For example, for the sorghum dataset, the metadata variable was composed of tissue type + tming and type of treatment, which could be “Leaves Preflowering Drought”, “Leaves Postflowering Drought”, “Leaves Control”, “Roots Preflowering Drought “, “Roots Postflowering Drought”, or “Roots Control”. Within a specific sample, the fraction of reads for a particular target is calculated as the number of reads with that anchor and target divided by the total number of reads with the anchor (and any target).

### 3.9 Plant Materials for Independent Validation

*A. thaliana* Col‐0 seeds were surface sterilized using 70% ethanol for 10 min, then plated on 0.5× Murashige and Skoog (MS) agar plates (PhytoTechnologies Laboratories) (pH = 5.7). Seeds were stratified at 4°C for 48 h in the dark, then plates were placed upright in growth chambers maintaining constant temperature of 23°C, with 150 μmol white light provided under long (16 h) day conditions. After 10 days of growth, seedlings  from individual plates were consolidated and immediately frozen and ground to a fine powder in liquid nitrogen before long‐term storage at −80°C.

### 3.10 RNA Extraction and cDNA Library Preparation

RNA was extracted from 100 mg of frozen *A. thaliana* tissues using the RNEasy Plant Mini Kit (Qiagen) per manufacturer recommendations. cDNA was synthesized from 2 μg RNA using M‐MLV Reverse Transcriptase (Thermo Scientific). DNA contamination was assessed by performing PCR amplification over a characterized intron junction and assessing intron retention (genomic DNA contamination) by agarose gel electrophoresis. Samples that produced a single band, of a size corresponding to properly spliced mRNA, were used for downstream analysis.

### 3.11 PCR and Amplicon Sequencing

For validation of splicing in AT1G13609, the region of interest was amplified from cDNA using DreamTaq master mix (Thermo Scientific). Primers as denoted in Figure S4A (5′ to 3′):

- Forward primer:GCGTAATTATGTCAGTGTTATTGGC
- Reverse primer:GCTTCTTCTCATCCAGTTTACAAGC

The resulting products were visualized on a 1.5% agarose gel, purified, and submitted to the NGS Amplicon‐EZ service from Azenta Life Sciences. The results were visualized using the Integrative genomics viewer (Robinson et al. 2011).

For genes where alternative splice forms could be separated through electrophoresis (AT1G79245), individual bands were purified separately and submitted for Sanger sequencing from Sequetech Corporation.

Primers as denoted in Figure S4B–D (5′ to 3′):

Primer_fw: GCCTGGAATCTGCACAAGTTG.

Primer_rev: TTACTGAAGTTATCATGGGAAGCACT.

## Author Contributions

J.S. and S.Y.R. conceived the project. E.M. analyzed data generated from SPLASH, worked on experimental validation of SPLASH results, and contributed to writing the manuscript. E.V.S. analyzed data generated from SPLASH, worked on experimental validation of SPLASH results, and contributed to writing the manuscript. M.K. contributed to development of the SPLASH method. B.X. analyzed data generated from SPLASH and contributed to writing the manuscript. S.D. contributed to development of the SPLASH method. S.Y.R. oversaw the project and contributed to writing the manuscript. J.S. oversaw the project, contributed to development of the SPLASH method, and contributed to writing the manuscript.

## Supporting information




**Figure S1**Overview of SPLASH method.
**Figure S2**Raw read counts for Figure 2 results.
**Figure S3**Raw read counts for Figure 3 results.
**Figure S4**Validation of SPLASH predictions.

**Table S1**Full list of GLM‐called anchors.

**Table S2**Metadata‐regulated anchors where one of top two targets does not align to reference genome.

**Data S1**Peer Review.

## Data Availability

All FASTQ files were downloaded from the Sequence Read Archive (SRA). The BioProject accession numbers for each dataset are *Arabidopsis* FLOE1 ‐ PRJNA704067 and PRJNA704079; *Arabidopsis* iron/phosphorus ‐ PRJNA685167; sorghum drought ‐ PRJNA527782; and maize pollen ‐ PRJNA732658 and PRJNA734295. The supplementary tables attached to the article contain 1) the full list of GLM‐called anchors (“Table S1” in the text) and 2) the metadata‐regulated anchors where one of the top two targets does not align to the reference genome (“Table S2” in the text). The full tables of significant anchors called by SPLASH for each dataset, and associated gene names, are available on Zenodo, along with the data used to generate the plots in the figures (Meyer et al. 2025). The supplementary tables on Zenodo referenced in the text contain the following: 1. **Supplementary Table A: “sorghum anchors**.” This table contains all significant anchors called by SPLASH from the sorghum dataset, along with the sorghum gene and position within the gene that the anchor maps to. 2. **Supplementary Table B “maize anchors.”** This table contains all significant anchors called by SPLASH from the maize dataset, along with the maize gene and position within the gene that the anchor maps to. 3. **Supplementary Table C: “*Arabidopsis* iron/phosphorus anchors.”** This table contains all significant anchors called by SPLASH from the *Arabidopsis* iron/phosphorus dataset, along with the *Arabidopsis* gene and position within the gene that the anchor maps to. 4. **Supplementary Table D: “*Arabidopsis* FLOE1 anchors.”** This table contains all significant anchors called by SPLASH from the *Arabidopsis* FLOE1 dataset, along with the *Arabidopsis* gene and position within the gene that the anchor maps to.
