# Supplementary material for: A Reference‐Free Algorithm Discovers Regulation in the Plant Transcriptome
Source: Plant Direct. 2026 Apr 9;10(4):e70061. doi: 10.1002/pld3.70061 (PMC13066497; doi:10.1002/pld3.70061)
Supplement: Supplementary file 1 — Figure S1Overview of SPLASH method. Figure S2Raw read counts for Figure 2 results. Figure S3Raw read counts for Figure 3 results. Figure S4Validation of SPLASH predictions. [file PLD3-10-e70061-s002.pdf]

Supplemental Figure 1

A

Is it designed to detect…

	Kmer-based pseudoalignment method e.g. Kallisto	Method for detecting new splice isoforms e.g. DiffSplice	De novo assembly from metagenomic sequence, e.g. PhyloFlash	SPLASH
**Regulated annotated alternative splicing?**	Yes	Yes	No	Yes
**Regulated splicing outside annotated gene boundaries?**	No	Yes	No	Yes
**Regulated expression of homologs missing from reference sequence?**	No	No	No	Yes
**Regulated changes in the associated microbiome?**	No	No	Yes	Yes

B

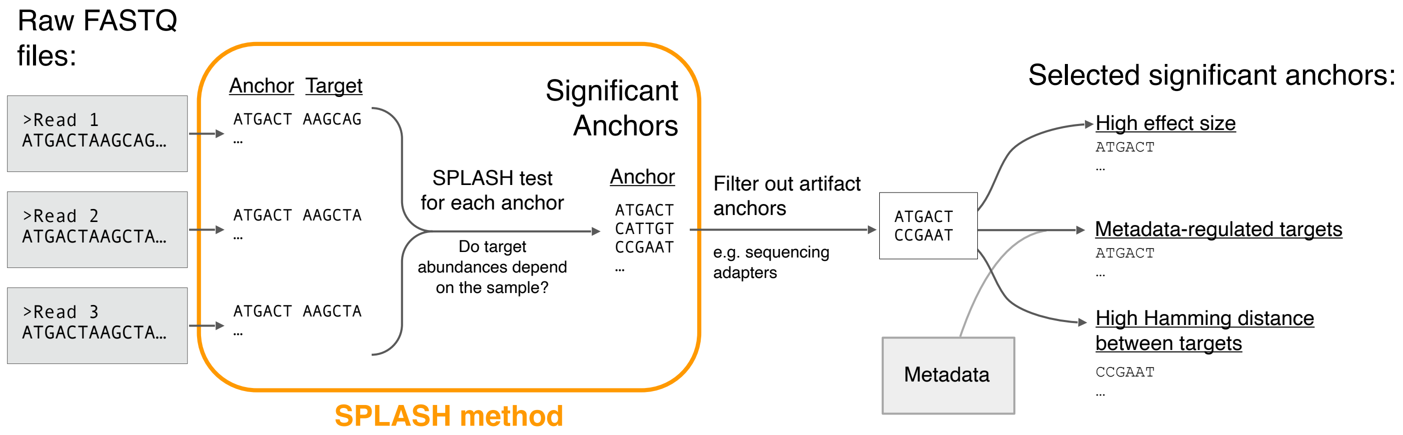

A. Comparison between SPLASH and other types of genomics analysis, showcasing the unique strengths of SPLASH. B: Overview of workflow for identifying anchors of interest. SPLASH takes in raw reads in FASTQ format and identifies “anchor” sequences (kmers) that precede different “target” sequences. For each such anchor, SPLASH runs a statistical test to determine whether the abundance of each target varies by sample. The significant anchors produced by SPLASH can then be prioritized by the user in various ways, such as selecting anchors with a large SPLASH effect size, selecting anchors where the target usage differs by metadata condition, selecting anchors with a high Hamming distance between targets, and more. See Methods for details.

Supplemental Figure 2

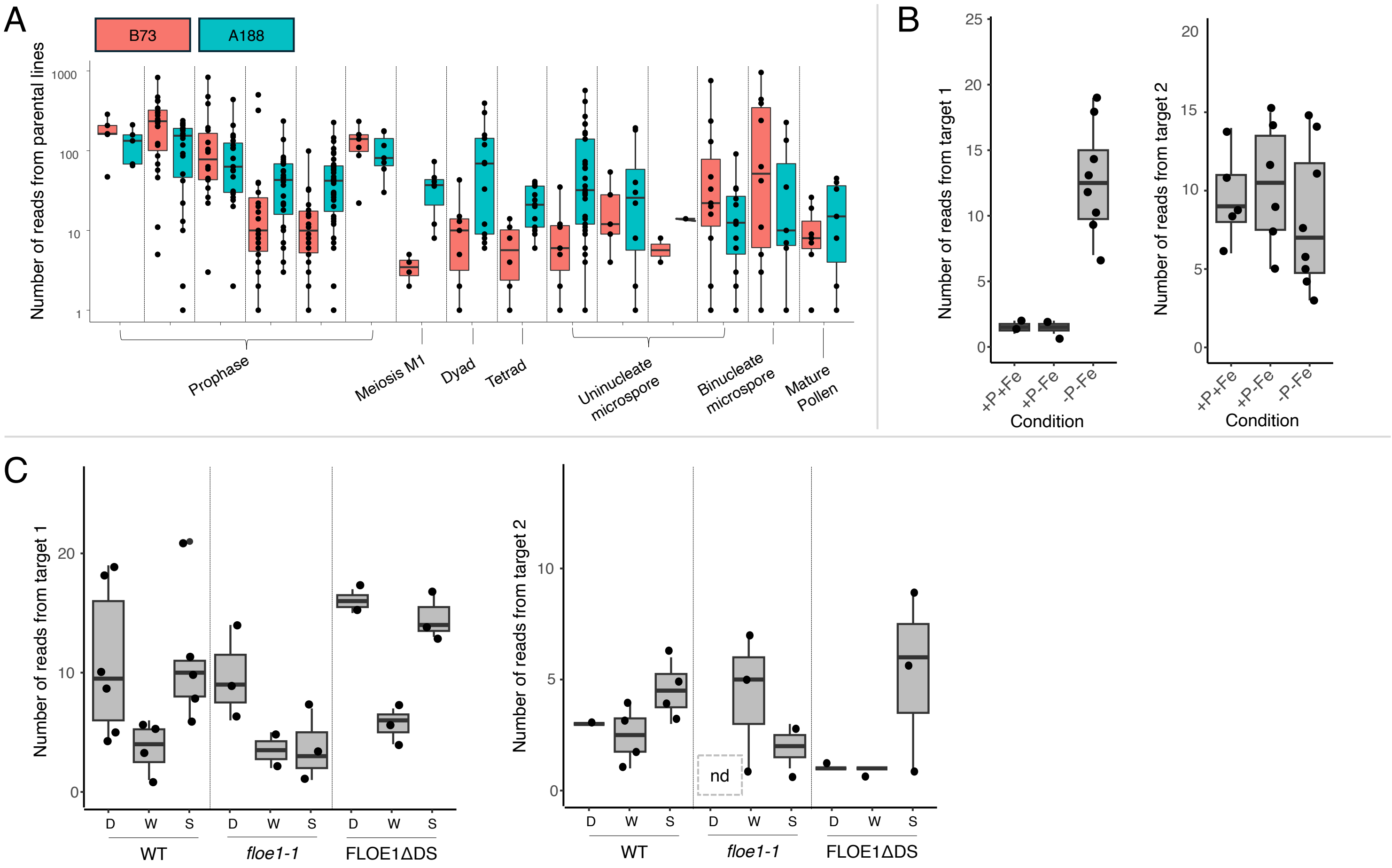

Number of raw reads within each sample from each of the top two targets. The top two targets typically represent the majority of reads even though most anchors have more than two targets. Horizontal lines in the boxes represent median; individual datapoints represent individual samples (single cells for maize pollen). A: Maize pollen dataset: the top two targets for this anchor align to alleles of Zm00001eb173470 from B73 and A188. The steps within the prophase label are, in order: leptotene with peripheral nucleolus, leptotene with central nucleolus, prezygotene, zygotene, pachytene, and interphase. Note: this anchor was only found in 260 out of 642 samples. B: *Arabidopsis* iron/phosphorus deprivation dataset: target 1 maps to a splice junction in AT1G74270 (ribosomal protein EL33Y), while target 2 includes the intron. -P-Fe indicates the phosphorus and iron doubly deprived condition; +P-Fe is only iron deprivation; and +P+Fe indicates no deprivation. C: *Arabidopsis* FLOE1 dataset: target 1 maps to an annotated splice junction between exons in AT2G36720, but target 2 maps to a cryptic splicing event from inside an intron to an exon. WT (wild type), *floe1-1* (FLOE1 deletion mutant), and FLOE1ΔDS (FLOE1 mutant with disordered region deleted) indicate the different seed genotypes; D (dry), W (wet), and S (salt) indicate the imbibition conditions for the seeds.

Supplemental Figure 3

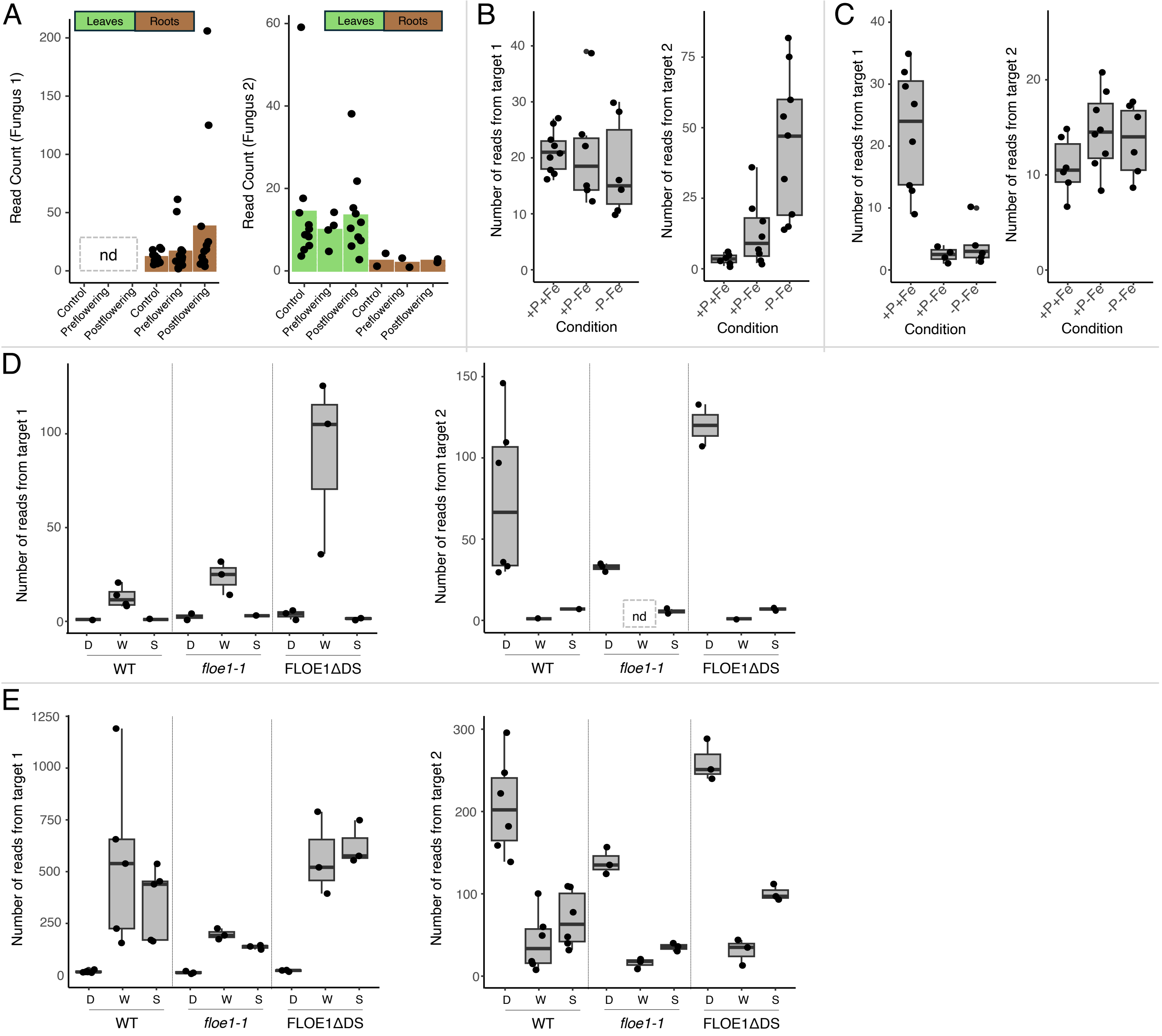

Number of raw reads within each sample from each of the top two targets. The top two targets typically represent the majority of reads even though most anchors have more than two targets. Horizontal lines in the boxes represent median; individual datapoints represent individual samples (single cells for maize pollen). A: Sorghum drought dataset: the top targets for this anchor BLAST to different fungal species. Target 1 had the best BLAST hits to fungal species in the genus *Alternaria*; the target 2 had the best BLAST hits to species in the genera *Pseudogymnoascus* and *Fusarium*. Tissue type is indicated by bar color. "Control" indicates samples with no drought stress; "preflowering" samples were droughted before the flowering stage; and "postflowering" samples were droughted after the flowering stage. N.d. means the anchor sequence was not found in the sequence data of samples in those conditions. This anchor was only found in 86 out of 198 total samples. B. *Arabidopsis* iron/phosphorus dataset: targets 1 and 2 for this anchor align to homologous genes AT3G08720 (protein kinase 19) and AT3G08730 (protein-serine kinase 6) respectively. -P-Fe indicates the phosphorus and iron doubly deprived condition; +P-Fe is only iron deprivation; and +P+Fe indicates no deprivation. C. *Arabidopsis* iron/phosphorus dataset: target 1 and target 2 for this anchor align to homologous genes AT1G62810 (copper amine oxidase 2) and AT3G43670 (copper amine oxidase 1) respectively. D: *Arabidopsis* FLOE1 dataset: targets 1 and 2 for this anchor align to homologous squalene monooxygenase genes, AT5G24160 and AT5G24150. WT (wild type), *floe1-1* (FLOE1 deletion mutant), and FLOE1ΔDS (FLOE1 mutant with disordered region deleted) indicate the different seed genotypes; D (dry), W (wet), and S (salt) indicate the imbibition conditions for the seeds. Note: this anchor was only found in 28 out of 36 samples. E. *Arabidopsis* FLOE1 dataset: targets 1 and 2 for this anchor align to homologous ERF/AP2 transcription factors, AT1G78080 (RAP2.4) and AT1G22190 (RAP2.4D).

Supplemental Figure 4

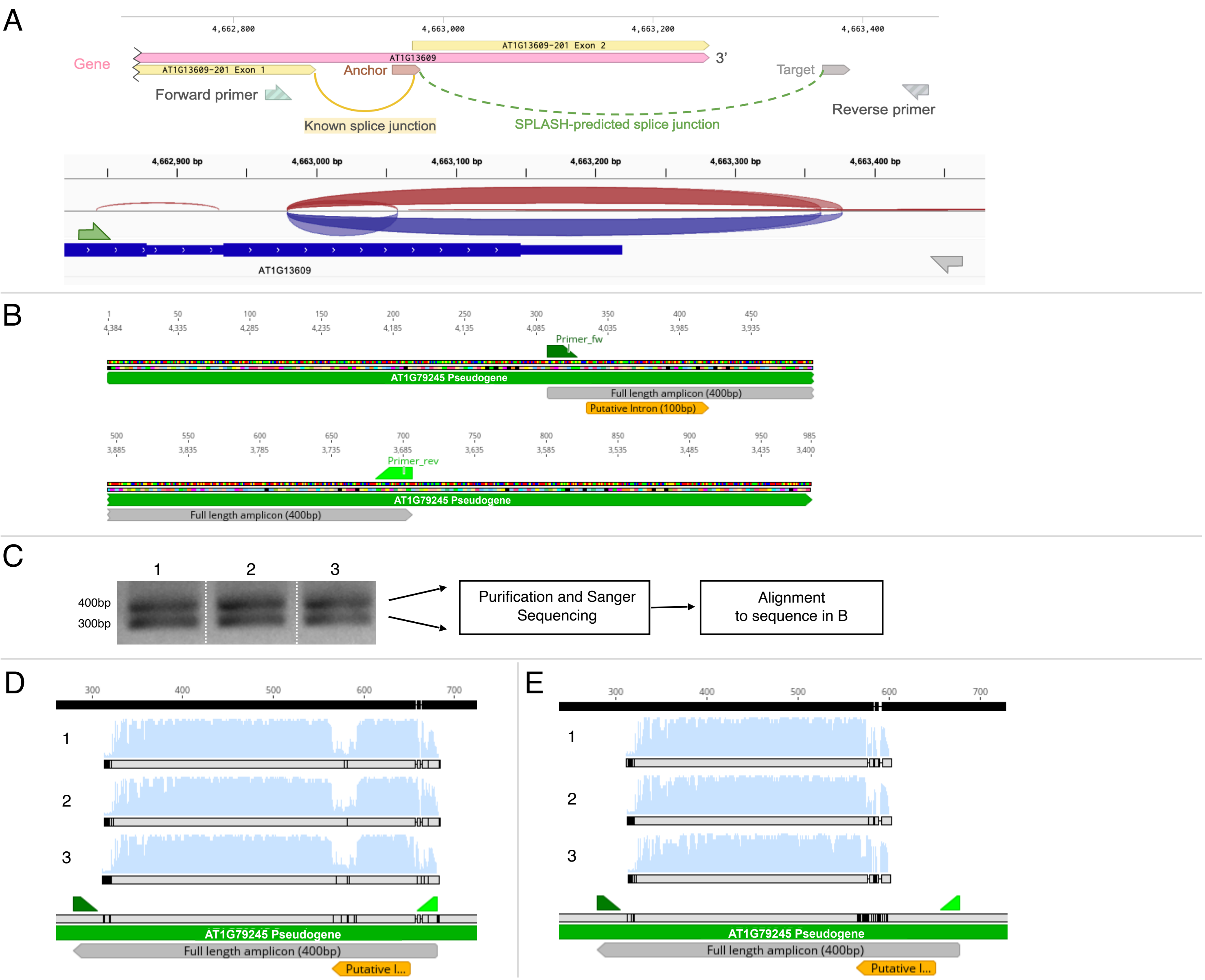

A: Validation of alternative splicing in AT1G13609. Above: SPLASH predicts a previously unannotated splice junction (dotted green line). Primers were designed surrounding this predicted junction. Below: The resulting PCR product was purified and sequenced via NGS Amplicon Sequencing (see Methods for details). The red and blue arcs represent reads with spliced alignments, confirming the presence of an unannotated splice junction spanning from the final exon of the gene to the 3' UTR region. B-D: Validation of alternative splicing in the AT1G79245 pseudogene. B: SPLASH annotates a 100bp putative intron in the *Arabidopsis* pseudogene AT1G79245. Nucleotide coordinates are provided above the sequence as grey numbers. The top row indicates positions within the represented 1kb fragment, starting at position 1. The bottom row indicates positions within the AT1G79245 pseudogene. AT1G79245 is encoded on the (-) strand of the *Arabidopsis* genome. Coordinates are provided according to the (+) strand of the genome, hence the descending position number. Predicted binding sites for Primer_fw and Primer_rev, calculated using the Primer_bind algorithm from Geneious, are shown as green trapezoids above the sequence. The predicted full length amplicon, and the position of the putative intron predicted by SPLASH are shown, with predicted 400bp (Intron retained) and 300bp (Intron spliced out) PCR products following amplification with Primer_fw and Primer_rev. C: Banding pattern on a 1% Agarose Gel following PCR amplification with Primer_fw and Primer_rev, from cDNA derived from 3 independent Col-0 seedling pools. Band sizes were estimated based on comparison to the GeneRuler 100bp ladder. A schematic of downstream analysis is shown in to the right of the visualized gel. Sanger Sequencing was performed using Primer_fw to prime the reaction. D-E: Alignments of Sanger Sequencing Results using DNA purified from the top (D) and bottom (E) bands shown in C. Per-nucleotide quality for samples 1-3 is shown as blue bars above the consensus sequence alignment. Alignment fidelity is shown as a grey bar for each sample, with dark lines or gaps indicating a failure to align, and light grey indicating a 100% sequence identity between samples.
