# Supplementary material for: A Reference‐Free Algorithm Discovers Regulation in the Plant Transcriptome
Source: Plant Direct. 2026 Apr 9;10(4):e70061. doi: 10.1002/pld3.70061 (PMC13066497; doi:10.1002/pld3.70061)
Supplement: Supplementary file 4 — Data S1Peer Review. [file PLD3-10-e70061-s004.pdf]

Plant Direct
Open Access

Title: A reference-free algorithm discovers regulation in the plant transcriptome

Authors: Elisabeth Meyer[1,2], Evan V. Saldivar[3,4], Marek Kokot[5], Bo Xue[4,6], Sebastian Deorowicz[5], Seung Y. Rhee[4,6], Julia Salzman[1,2,7,8] *

Decision Letter Round 1:

November 29, 2024

Julia Salzman
Stanford University
Stanford, California

RE: A reference-free algorithm discovers regulation in the plant transcriptome

Dear Dr. Salzman:

Thank you for submitting to Plant Direct. All required reviews have been returned and we have now finished our evaluation of your manuscript. In light of the reviewers' and editor's comments, further revisions are needed before the paper can be accepted for publication in Plant Direct.

Please view the editors' and reviewers' comments below and use their suggestions as a guide while you work on your revision.

When uploading the revised version of this article, please be sure to include the following:

-A word document that contains your response to the reviewers. You should respond to each reviewer comment and note the changes made to the manuscript. If you do not agree with a reviewer's comment and choose not to make a suggested revision, please explain why. Please try to provide as complete an answer as possible to each reviewer's criticisms in the "response to reviewers" section.
-A tracked changes document
- A clean version of the latest version of the manuscript

To upload your revision, please click the link below.
https://plantdirect.msubmit.net/cgi-bin/main.plex?el=A7Lr7jb4A6ouO3I5A9ftdyq8pXCY3QqOP8HUM3n0s6gZ

In order to provide as timely a service as possible, we ask that your revision is resubmitted within three months after receipt of this request. If an extension is needed, please send a request, along with a brief explanation, to the editorial office at plantdirect@wiley.com .

Thank you very much for giving us an opportunity to review your work. I look forward to receiving the next version.

Sincerely,
Jyothilakshmi Vadassery

-- Editor comments:

-- Reviewer comments:
Reviewer #1:

The manuscript, "A reference-free algorithm discovers regulation in the plant transcriptome" aimed to analyze transcriptional and post-transcriptional regulation from RANseq data using the SPLASH algorithm. The algorithm is suggested to discover differential homolog expression under different experimental setups as well as genetic backgrounds. Moreover, SPLASH enabled the discovery of differential gene regulation, alternative splicing, cryptic splicing, as well as previously unannotated splice junctions.

The manuscript needs more clarity and explanation to make the narrative clear to the readers. Some of the observations may also require validation to highlight the biological reliability of the SPLASH-driven study of genomic or transcriptomic regulation. Moreover, comparisons with the existing methods based on reference for the quality of output, resolution, and computational requirements may also be highlighted. A proper explanation of the logic of Kmers i.e. anchors and how it works may make the method more relatable.

Calling the method "reference-free" is a bit ambiguous, as eventually the anchor/target sequence is aligned to the genome. Therefore, the identification of a significant anchor is essentially reference-free and subsequently requires a genome for biological interpretation.

I was also wondering if the purpose is to identify differential gene regulation, differential homolog expression, alternative splicing, cryptic splicing, as well as previously unannotated splice junctions from a

plant species with available genome or if the purpose is to get insights into transcriptomic and genomic regulation of a species without a reference or both. This would require clarification.

If the purpose is to get novel insights from an available genome, how does the performance compare with alignment- or de novo assembly-driven analysis? If the purpose is to use the algorithm for a plant species without reference genome or transcriptome, possibly an example of a non-model plant species with limited transcriptome data should be considered.

Unaligned anchors make use of reference genome, thus will not be applicable for plants without reference genome

A detailed flow chart/workflow with branches showing different analyses/usage with the steps would help in better understanding.

Computational requirements, particularly RAM, storage, CPU usage, and run time, may also be explained to suggest the requirements at the user end. Also, the possibility of using multiple threads and speed of processing the data (number of reads) may be provided and compared with the alignment- and assembly-based methods.

In addition to the validation of previously unannotated splice junctions, the possibility of validation of differential homolog expression and cryptic splicing may also be considered for the reliability of the performance of the algorithm.

Supplementary tables are not labeled and also without the table legends which makes the tables difficult to understand. Overall organization of the manuscript makes it difficult to follow.

Reviewer #2:

The manuscript introduces SPLASH, a reference-free algorithm designed to detect regulatory events in plant transcriptomes by analyzing RNA-seq data without aligning reads to a reference genome. SPLASH demonstrates its utility by analyzing datasets from maize, Arabidopsis, and sorghum, uncovering novel biological insights such as alternative splicing, cryptic splicing, and condition-dependent homolog expression. The study highlights SPLASH's potential to address challenges in transcriptome analysis for species with incomplete or complex genomes. However, several issues need to be addressed before the manuscript can be considered for publication.
Major Concerns
1. Comparative Analysis with Existing Tools:
The manuscript lacks a systematic comparison between SPLASH and widely used reference-based tools. Without benchmarking, it is difficult to evaluate SPLASH's relative accuracy, sensitivity, and capability to uncover novel findings.

2. Biological Context of Findings:
The manuscript describes several novel regulatory phenomena but does not sufficiently explore their biological implications. For example, cryptic splicing and differential homolog regulation are intriguing discoveries that warrant deeper discussion of their potential functional roles in plant biology.
3. Scalability and Practical Application:
SPLASH's scalability to larger datasets or highly polyploid genomes is not thoroughly addressed. While parameters and runtime details are mentioned, additional evidence demonstrating its efficiency and applicability to diverse plant species with complex transcriptomes is needed.
4. Clarity and Accessibility for Non-Computational Biologists:
The technical descriptions of SPLASH and its statistical framework are dense and may be inaccessible to readers without computational expertise. Simplifying explanations and providing more context on statistical methods would make the manuscript more inclusive.
5. Algorithm Constraints:
SPLASH's current limitation in processing paired-end reads is a notable drawback. While this is acknowledged in the methods, a discussion of how it might affect the interpretation of results or plans for addressing this limitation in future iterations would enhance the manuscript.
Minor Suggestions
1. Introduction: Provide a concise overview of other reference-free or alignment-free methods, emphasizing SPLASH's novelty. Elaborate on the specific challenges SPLASH addresses in plant transcriptomics.
2. Methods: Expand details on how SPLASH handles noise and sequencing artifacts.
3. Results: Discuss the significance of discoveries, particularly cryptic splicing and homolog regulation, in biological terms.
4. Discussion: Reflect on SPLASH's limitations, such as its inability to process paired-end reads, scalability concerns, and reliance on curated datasets. Discuss potential integrations with alignment-based tools for hybrid analyses. Address future directions for improving SPLASH, particularly for diverse plant genomes.

Decision Letter Round 2:

February 21, 2025

Julia Salzman
Stanford University
Stanford, California

RE: A reference-free algorithm discovers regulation in the plant transcriptome

Plant Direct
Open Access

Dear Dr. Salzman:

Thank you for submitting to Plant Direct. All required reviews have been returned and we have now finished our evaluation of your manuscript. In light of the reviewers' and editor's comments, further minor revisions are needed before the paper can be accepted for publication in Plant Direct.

Please view the editors' and reviewers' comments below and use their suggestions as a guide while you work on your revision.

When uploading the revised version of this article, please be sure to include the following:

-A word document that contains your response to the reviewers. You should respond to each reviewer comment and note the changes made to the manuscript. If you do not agree with a reviewer's comment and choose not to make a suggested revision, please explain why. Please try to provide as complete an answer as possible to each reviewer's criticisms.
-A tracked changes document with each change highlighted
- A clean version of the latest version of the manuscript

In general both the reviewers are satisfied with the revision. Please see the reviewer's comments after revision. Reviewer 2 has some important points that you can address before the final acceptance.

Please discuss in detail why genomic alignment represents an important step in the interpretation of the discovery.

What kind of interpretation would be possible without using a reference genome. Some case study with a small analysis would enrich the MS

To upload your revision, please click the link below.
https://plantdirect.msubmit.net/cgi-bin/main.plex?el=A4Lr4jb4B5ouO6I5A9ftdyq8pXCY3QqOP8HUM3n0s6gZ

In order to provide as timely a service as possible, we ask that your revision is resubmitted within three months after receipt of this request. If an extension is needed, please send a request, along with a brief explanation, to the editorial office at plantdirect@wiley.com .

Please note that, in addition to publishing reviewer comments, the author's responses to review comments will also be published alongside the final version of the paper. If you would not like the author's responses to be published, please contact the editorial office at plantdirect@wiley.com .

Thank you very much for giving us an opportunity to review your work. I look forward to receiving the next version.

Sincerely,

Jyothilakshmi Vadassery

Gustavo MacIntosh

Editor, Plant Direct

-- Editor comments:
In general both the reviewers are satisfied with the revision. Please see the reviewer's comments after revision. Reviewer 2 has some important points that you can address before the final acceptance.

Please discuss in detail why genomic alignment represents an important step in the interpretation of the discovery.

What kind of interpretation would be possible without using a reference genome. Some case study with a small analysis would enrich the MS

-- Reviewer comments:
Reviewer #1:

The authors have referred to their previous publication on SPLASH to explain several comments of both the reviewers related to the quality of output, resolution, computational requirements, and comparison with the existing methods, which is good. The previous publication has established the SPLASH algorithm as an efficient method without the need for reference genomes using data from viruses, human, and lemur as well as non-model organisms. They follow up with testing and analyzing SPLASH for plant transcriptomes using Arabidopsis, maize, and sorghum data in the current manuscript.

While some of the outcome and performance would be expected based on their previous work, some of the analyses, in particular, usage of non-model plant data without a reference genome (like they did using Octopus and eelgrass in their previous publication) would certainly make the analysis using plant systems more compelling and convince the users working with plant systems for the usage of SPLASH with such systems.

Further, the authors suggest that the genomic alignment represents an important step in the interpretation of the discovery. I could not find much explanation in this context in the revised

manuscript as mentioned in the response document, except for the addition of one sentence. However, since the authors call the algorithm reference-free, what kind of interpretation would be possible without using a reference genome. Some kind of case study would certainly help in this direction.

Reviewer #2:

The authors addressed all the comments from the previous review and made all the required changes.

Decision Letter Round 3:

March 8, 2025
Julia Salzman
Stanford University
Stanford, California

MSID: 2024-01581R2
MS TITLE: A reference-free algorithm discovers regulation in the plant transcriptome

Dear Dr. Julia Salzman:

I am pleased to inform you that your manuscript "A reference-free algorithm discovers regulation in the plant transcriptome" has been accepted for publication in Plant Direct.

Your article will appear online in the next available issue of Plant Direct. To ensure your article gets published as quickly as possible, please pay attention to the steps detailed below. We have found that most of the delays happen at this stage, especially at the payment stage, so please respond as quickly as possible when prompted.

License Agreement: Once your article has been accepted it will move to Production and undergo admin and file checking - you may receive an email with any queries we have at this stage. When all required items are received by the publisher and queries resolved, the corresponding author will receive an email from Wiley's Author Services system which will ask them to log on at https://authorservices.wiley.com/bauthor and will present them with the appropriate license for completion. Your article cannot be published until both the signed license agreement and payment of the article fee have been received.

Plant Direct
Open Access

Payment of the Open Access Article Publication Fee: All articles published in Plant Direct are fully open access: immediately and freely available to read, download and share. Plant Direct charges a publication fee to cover the publication costs. The corresponding author for this manuscript should have already received a quote from the payments team (cs-openaccess@wiley.com) with the estimated article publication fee; please email cs-openaccess@wiley.com if this has not been received. The corresponding author should log on to the Wiley Author Services site, where the publication fee can be paid by credit card or an invoice. Pro Forma can also be requested. Payment of the publication charge must be received before the article will be published online.

Proofs: You will have the opportunity to look over your paper once more when you receive the author proofs for your article. The proofs will be with you in approximately two weeks. Please note that, in addition to publishing reviewer comments, the author's responses to review comments will also be published alongside the final version of the paper. If you would not like the author's responses to be published, please contact the editorial office at plantdirect@wiley.com .

Promotion of your article: You can help your research get the attention it deserves! Check out Wiley's free Promotion Guide for best-practice recommendations for promoting your work at www.wileyauthors.com/eeo/guide. And learn more about Wiley Editing Services which offers professional video, design, and writing services to create shareable video abstracts, infographics, conference posters, lay summaries, and research news stories for your research at www.wileyauthors.com/eeo/promotion.

Thank you again for your contribution to Plant Direct. If you have any questions, feel free to contact the editorial office at plantdirect@wiley.com .

Sincerely,

Jyothilakshmi Vadassery

Gustavo MacIntosh

Editor, Plant Direct

Response to Reviewer Round 1:

We greatly appreciate the reviewer's time and effort in providing constructive feedback on our revised manuscript.

Our responses to the reviewer comments are in bold.

Plant Direct
Open Access

Reviewer #1:

The manuscript, "A reference-free algorithm discovers regulation in the plant transcriptome" aimed to analyze transcriptional and post-transcriptional regulation from RANseq data using the SPLASH algorithm. The algorithm is suggested to discover differential homolog expression under different experimental setups as well as genetic backgrounds. Moreover, SPLASH enabled the discovery of differential gene regulation, alternative splicing, cryptic splicing, as well as previously unannotated splice junctions.

The manuscript needs more clarity and explanation to make the narrative clear to the readers. Some of the observations may also require validation to highlight the biological reliability of the SPLASH-driven study of genomic or transcriptomic regulation. Moreover, comparisons with the existing methods based on reference for the quality of output, resolution, and computational requirements may also be highlighted. A proper explanation of the logic of Kmers i.e. anchors and how it works may make the method more relatable.

We thank the reviewer for their thoughtful evaluation and constructive feedback. We have included orthogonal validation of biological results where it was feasible (i.e. in Arabidopsis). We do not have the resources to carry out validation of results in sorghum and maize. The quality of output, resolution, and computational requirements of SPLASH are outlined in the original SPLASH publication (http://dx.doi.org/10.1016/j.cell.2023.10.028) which is also referenced in the text. We refrained from very technical explanations of the method because the method has already been published, and because we would like this manuscript to reach a wider audience who would be more interested in the method's potential for biological discovery.

Calling the method "reference-free" is a bit ambiguous, as eventually the anchor/target sequence is aligned to the genome. Therefore, the identification of a significant anchor is essentially reference-free and subsequently requires a genome for biological interpretation.

We agree that the genomic alignment represents an important step in interpretation of the discovery. However, a genome is not strictly necessary, as a transcriptomic sequence can be identified on the basis of similarity e.g. using a tool like BLAST. We will clarify this terminology in the text.

I was also wondering if the purpose is to identify differential gene regulation, differential homolog expression, alternative splicing, cryptic splicing, as well as previously unannotated splice junctions from a plant species with available genome or if the purpose is to get insights into transcriptomic and genomic regulation of a species without a reference or both. This would require clarification.

If the purpose is to get novel insights from an available genome, how does the performance compare with alignment- or de novo assembly-driven analysis? If the purpose is to use the algorithm for a plant species without reference genome or transcriptome, possibly an example of a non-model plant species with limited transcriptome data should be considered.

SPLASH can enable discovery both in species with a well-characterized genome and in species with no genome assembly. We will clarify this in the text. The performance of SPLASH has been outlined in the original SPLASH publication (http://dx.doi.org/10.1016/j.cell.2023.10.028).

Unaligned anchors make use of reference genome, thus will not be applicable for plants without reference genome

Although we did use the reference genomes in this manuscript, a genome is not strictly necessary for identifying unaligned anchors. Instead, a transcriptomic sequence can be identified on the basis of similarity e.g. using a tool like BLAST. We will explain this in the text.

A detailed flow chart/workflow with branches showing different analyses/usage with the steps would help in better understanding.

Computational requirements, particularly RAM, storage, CPU usage, and run time, may also be explained to suggest the requirements at the user end. Also, the possibility of using multiple threads and speed of processing the data (number of reads) may be provided and compared with the alignment- and assembly-based methods.

The method steps, computational requirements, and parallel processing of SPLASH are all discussed in the original SPLASH publication (http://dx.doi.org/10.1016/j.cell.2023.10.028).

In addition to the validation of previously unannotated splice junctions, the possibility of validation of differential homolog expression and cryptic splicing may also be considered for the reliability of the performance of the algorithm.

We agree that validation of differential homolog expression and cryptic splicing would be ideal. Unfortunately, this would require replicating the studies from which we obtained the data, which would be extremely resource-intensive and beyond the scope of this study.

Supplementary tables are not labeled and also without the table legends which makes the tables difficult to understand. Overall organization of the manuscript makes it difficult to follow.

We appreciate this comment and will add information to clarify the contents of the supplementary tables.

Reviewer #2:

The manuscript introduces SPLASH, a reference-free algorithm designed to detect regulatory events in plant transcriptomes by analyzing RNA-seq data without aligning reads to a reference genome. SPLASH demonstrates its utility by analyzing datasets from maize, Arabidopsis, and sorghum, uncovering novel biological insights such as alternative splicing, cryptic splicing, and condition-dependent homolog expression. The study highlights SPLASH's potential to address challenges in transcriptome analysis for species with incomplete or complex genomes. However, several issues need to be addressed before the manuscript can be considered for publication.
Major Concerns
1. Comparative Analysis with Existing Tools:

The manuscript lacks a systematic comparison between SPLASH and widely used reference-based tools. Without benchmarking, it is difficult to evaluate SPLASH's relative accuracy, sensitivity, and capability to uncover novel findings.

We thank the reviewer for their thoughtful evaluation and constructive feedback. The reliability, precision, and computational requirements of SPLASH are outlined in the original SPLASH publication (http://dx.doi.org/10.1016/j.cell.2023.10.028) which is also referenced in the text. We refrained from very technical explanations of the method because the method has already been published, and because we would like this manuscript to reach a wider audience who would be more interested in the method's potential for biological discovery.

2. Biological Context of Findings:
The manuscript describes several novel regulatory phenomena but does not sufficiently explore their biological implications. For example, cryptic splicing and differential homolog regulation are intriguing discoveries that warrant deeper discussion of their potential functional roles in plant biology.

We agree that the cryptic splicing and differential homolog regulation are interesting findings worthy of further investigation. In the case of cryptic splicing in Arabidopsis, we discussed possible causes for this event, including the presence of an upstream open reading frame. We will include some additional discussion on potential roles of differential homolog regulation.

3. Scalability and Practical Application:
SPLASH's scalability to larger datasets or highly polyploid genomes is not thoroughly addressed. While parameters and runtime details are mentioned, additional evidence demonstrating its efficiency and applicability to diverse plant species with complex transcriptomes is needed.

We agree that the study of large and highly polyploid genomes is an important challenge in plant transcriptomics. We chose maize as an example organism with high levels of gene duplication. Our results are meant as an illustration of the uses of SPLASH, and are certainly not exhaustive. Unfortunately, we do not have the capabilities at this point to process large amounts of additional data.

4. Clarity and Accessibility for Non-Computational Biologists:
The technical descriptions of SPLASH and its statistical framework are dense and may be inaccessible to readers without computational expertise. Simplifying explanations and providing more context on statistical methods would make the manuscript more inclusive.

We appreciate this criticism and understand the challenge of presenting a method to a wide audience with a variety of different backgrounds. We note that Reviewer 1 requested additional technical details, while Reviewer 2 was concerned with the technical descriptions being too dense. We have tried to strike a balance between a technical, methods-focused manuscript and a manuscript centered on the interesting biology being discovered.

5. Algorithm Constraints:
SPLASH's current limitation in processing paired-end reads is a notable drawback. While this is acknowledged in the methods, a discussion of how it might affect the interpretation of results or plans for addressing this limitation in future iterations would enhance the manuscript.

We thank the reviewer for this insightful comment. There are statistical subtleties to using both pairs in paired end reads that have prevented us from including both reads in SPLASH as we want to keep the algorithm completely statistical. We believe that this is an interesting but highly technical subject that is beyond the scope of this plant-focused biological paper. In the future, we anticipate SPLASH will be available for long read data which will significantly enhance inference possible with SPLASH.

Response to Reviewer Round 2:

We greatly appreciate the reviewers' time and effort in providing constructive feedback on our revised manuscript.

Our responses to the reviewer comments are in bold.

Please discuss in detail why genomic alignment represents an important step in the interpretation of the discovery.

For almost all the examples in this manuscript, genomic alignment was very important as it was used to identify the genes involved, and to interpret the type of event (e.g. expression differences by allele or alternative splicing). A short explanation has been added to each example in the main text to clarify the role of genomic alignment in the interpretation of the SPLASH discovery. In addition, some clarifying sentences have been added to the introduction and conclusion.

What kind of interpretation would be possible without using a reference genome. Some case study with a small analysis would enrich the MS

Without a reference genome, interpretation is still possible. One simple approach is to use a sequence database such as BLAST to identify the sequences or closely related sequences. In fact, this approach was used to identify fungal transcripts in field-droughted sorghum samples (see the Supplementary Information section). When multiple species are present in a sample, alignment to a single genome does not provide sufficient information. Several sentences have been added throughout the manuscript to clarify this point, including in the fungal example.
